# Mobile Health Technology for Personalized Tobacco Cessation Support Among Cancer Survivors and Caregivers in Laos (Project SurvLaos): Protocol for a Pilot Randomized Controlled Trial

**DOI:** 10.2196/66517

**Published:** 2025-07-30

**Authors:** Phayvanh Keopaseuth, Phonepadith Xangsayarath, Shweta Kulkarni, Khatthanaphone Phandouangsy, Chanthavy Soulaphy, Phetsamone Alounlungsy, Vangnakhone Dittaphong, Dalouny Xayavong, Champadeng Vongdala, Latsamy Siengsounthone, Michael Businelle, Summer G Frank-Pearce, Damon J Vidrine, Jennifer I Vidrine, Thanh Cong Bui

**Affiliations:** 1 Ministry of Health of Lao People's Democratic Republic Vientiane Capital Lao People's Democratic Republic; 2 Department of Biostatistics and Epidemiology Hudson College of Public Health University of Oklahoma Health Sciences Oklahoma City, OK United States; 3 Secretariat of the National Tobacco Control Committee Vientiane Capital Lao People's Democratic Republic; 4 National Center for Laboratory and Epidemiology Ministry of Health of Lao PDR Vientiane Capital Lao People's Democratic Republic; 5 Lao National Cancer Center Ministry of Health of Lao PDR Vientiane Capital Lao People's Democratic Republic; 6 Setthathirath Hospital Vientiane Capital Lao People's Democratic Republic; 7 TSET Health Promotion Research Center Stephenson Cancer Center University of Oklahoma Health Sciences Oklahoma City, OK United States; 8 Department of Family and Preventive Medicine College of Medicine University of Oklahoma Health Sciences Oklahoma City, OK United States; 9 Department of Health Outcomes and Behavior Moffitt Cancer Center Tampa, FL United States

**Keywords:** smoking cessation, tobacco cessation, tobacco dependence, cancer survivorship, mHealth, Lao People’s Democratic Republic

## Abstract

**Background:**

Tobacco use remains a major cause of preventable deaths and evidence suggests that smoking cessation offers considerable benefits for patients with and survivors of cancer. In the Lao People’s Democratic Republic (Lao PDR), approximately 60% of male patients and 15% of female patients with cancer smoke cigarettes. Nevertheless, there is no tobacco treatment program for this population.

**Objective:**

This pilot randomized controlled trial (RCT) aims to evaluate the feasibility and preliminary efficacy of our mobile health–based automated treatment (AT) program to help Lao cancer survivors and caregivers quit smoking cigarettes.

**Methods:**

We used an intervention mixed methods research design, which included a pilot 2-group interventional RCT and an embedded qualitative component to explain RCT outcomes. In the pilot RCT, cancer survivors or caregivers (n=80, no dyads) who smoked were recruited from national hospitals in Vientiane. Recruited participants were randomized to 1 of 2 treatment groups: standard care (SC) or AT. SC consisted of brief advice to quit smoking delivered by research staff, self-help written materials, and a 2-week supply of nicotine replacement therapy (transdermal patches). AT consisted of all SC components plus our fully automated, interactive, smartphone-based behavioral treatment program personalized and tailored to cancer survivors or caregivers and delivered by our Insight app. Feasibility outcomes of interest include the percentages of intervention messages delivered and viewed, and participant retention at the 3-month follow-up. The preliminary efficacy outcome is biochemically confirmed self-reported 7-day point prevalence abstinence at 3 months post study enrollment. During the interventional RCT and after the 3-month follow-up assessment, we used additional open-ended questions to explore why and how the participants did or did not successfully quit smoking and stay abstinent.

**Results:**

Data collection occurred from April 2022 to May 2023. Outcome analyses are ongoing, and results are expected to be published in 2025.

**Conclusions:**

Our course of research will address the critical need of having a scalable and sustainable tobacco cessation treatment program for patients with cancer and their caregivers in Lao PDR. The preliminary data from this pilot project will lay a foundation for a subsequent fully powered RCT to evaluate the actual efficacy of our mobile health–based AT program. Ultimately, our course of research will contribute to reducing tobacco-related complications in cancer treatments, comorbidities, tobacco-related cancer recurrence, and mortality rates in Lao PDR.

**Trial Registration:**

ClinicalTrials.gov NCT05253573; https://clinicaltrials.gov/study/NCT05253573

**International Registered Report Identifier (IRRID):**

DERR1-10.2196/66517

## Introduction

Globally, tobacco is responsible for around 6 million deaths annually; 80% of these occur in low- and middle-income countries (LMICs) [1,2]. Tobacco use is the primary modifiable risk factor for cancer prevention and remains a leading cause of preventable deaths [1]. Cigarette smoking prevention and cessation stand out as the most impactful and cost-effective interventions among various evidence-based preventive health services [3]. Despite this critical need, smoking cessation treatments in LMICs are frequently unavailable or unaffordable for the majority of the population [1].

A report of cancer incidence and mortality estimates in 2022 indicated that the Lao People’s Democratic Republic (Lao PDR) has the largest age-standardized cancer mortality rate per 100,000 person-years in Southeast Asia [4]. The same report also showed that more than 50% of the new cancer cases (n=9101) in Lao PDR resulted in mortality (6215 cancer deaths). Tobacco smoking prevalence among patients with cancer in Lao PDR is high: 60% in men and 15% in women in our preliminary analysis of data from the Lao National Cancer Center (LNCC) medical records, and up to 87% in patients with lung cancer [5]. Data regarding smoking prevalence among caregivers of Lao patients with cancer are lacking. However, the most recent national survey of the general Lao population indicated that 51% of adult men and 7% of adult women smoke tobacco [4,5], suggesting a potentially equivalent smoking prevalence among caregivers.

Evidence from high-income countries suggests that smoking cessation offers considerable benefits for cancer survivors, including improving cancer treatment outcomes, reducing recurrence rates, reducing second primary tumor development, and reducing overall cancer mortality rates [6,7]. Thus, developing and evaluating a scalable and sustainable tobacco cessation treatment program is essential to improve cancer treatment and survivor outcomes. In addition, for caregivers who smoke, seeing their family members, relatives, or friends combating tobacco-induced cancer and mortality is a teachable moment for the caregivers to quit smoking to protect their own health. When caregivers quit, the likelihood that patients with or survivors of cancer are exposed to second-hand smoking and craving triggers is also reduced; therefore, the journey to quitting smoking among patients with cancer will be more likely to succeed. Despite this need, no widely accessible tobacco treatment programs are available in major (public) hospitals in Lao PDR, including cancer hospitals or related institutions, as confirmed in our discussions with leaders of the LNCC and the National Tobacco Control Committee (NTCC) of Lao PDR. A quit line offering telephone counseling was piloted at the national Mahosot Hospital; however, funding limitations hinder the NTCC’s ability to train and retain more counselors for nationwide implementation. Therefore, there is a critical need for an evidence-based and highly scalable tobacco cessation treatment in Lao PDR to prevent smoking-related morbidity and mortality, especially among cancer survivors and caregivers.

The World Health Organization (WHO) acknowledges that mobile health (mHealth) could transform the face of health service delivery across the globe, including delivery in the lowest-income countries [8]. Data from the Lao government’s Statistics Bureau show that 96% of Laos’ population subscribed to a mobile phone plan in 2022 [9]. Although Laos is among the lowest-income countries [10], 100% of the Lao urban districts and 96% of the rural villages are covered by a mobile-cellular network [9]. These provide an ideal yet largely untapped mechanism to deliver smoking cessation treatment in Lao PDR. A small but growing body of research indicates that mHealth interventions are feasible in Lao PDR [11-13]. The Lao Ministry of Health’s (MOH’s) receptiveness and support of mHealth solutions in these studies increase the potential for sustainability and widespread adoption of our proposed mHealth intervention approach. The effectiveness of smoking cessation interventions using text messaging, traditionally delivered via SMS, is shown in both randomized controlled trials (RCT) and a recent Cochrane review [14,15]. Mobile phone–delivered text messaging has been identified as one of the most affordable interventions [16] and has been endorsed and used by several international organizations, including the WHO, in their global tobacco control efforts [17,18]. Many agencies, such as the US National Cancer Institute (NCI) have made scientifically supported apps available for public use [19].

We previously developed a theoretically and empirically based mHealth smoking cessation intervention and pilot-tested it with 50 Cambodian people with HIV who smoked cigarettes [20]. Our results have shown that our proposed mHealth approach is highly feasible, appropriate, and potentially scalable for LMIC settings. This mHealth automated treatment (AT) program includes a fully automated, interactive, personalized, smartphone-based intervention for behavioral treatment, delivered through our Insight platform. In the parent project of this study (R21CA253600), we have adapted and validated the AT content to the sociocultural context, language, and communication styles of Lao people in the general population.

Herein, we describe the design, methods, and analysis plans for a supplemental pilot project that focuses on patients with and survivors of cancer, funded by an Administrative Supplement grant (3R21CA253600-02S1) from the US NCI. This pilot project aims to adapt and evaluate the feasibility and preliminary efficacy of our mHealth-based AT intervention to help Lao patients with and survivors of cancer and their caregivers quit smoking cigarettes. We used an intervention mixed methods research design, which included a 2-group interventional RCT and an embedded qualitative component to explain RCT outcomes. Findings from this pilot RCT will provide useful information for larger, fully powered RCTs to assess the efficacy and effectiveness of mHealth-based smoking cessation treatments and ultimately to reduce tobacco-related morbidities and mortalities in Lao PDR.

## Methods

### Ethical Considerations

The study was approved by the institutional review boards (IRBs) of the Lao MOH–National Ethics Committee for Health Research (NECHR; IRB00006227) and of The University of Oklahoma Health Sciences Center (OUHSC; IRB#: 14125). Our collaborative hospitals (where the study took place)—Setthathirath Hospital (SH) and LNCC—are public hospitals under the management of the Lao MOH and have assurances relying upon the NECHR IRB. All participants underwent an informed consent process and provided written informed consent to participate. Participants were informed of the nature of the investigation, the steps that they would complete, and the types of interventions involved. Specifically, for the RCT, potential participants were made aware that (1) they would be participating in a research study, (2) they would be asked to complete assessments according to research protocols, (3) they would have a chance of being randomized to either treatment arm of the study, and (4) their study data, without any identifiers, will be posted on ClinicalTrials.gov. Participants received explanations about the potential risks of participation, measures to protect against risks, and the alternative option of not participating. The whole informed consent process was conducted in Lao language by local research staff. Participant privacy and confidentiality have been carefully maintained. All study paper records with personal identifiers were maintained in locked offices and locked file cabinets at the participating institutions. Electronic files and data are stored on secure OUHSC servers, following information security standards required by the OUHSC Information Technology Department and IRB. Only research staff have access to these paper records or electronic data. All data will be analyzed and reported in aggregate form, and individual participants will not be identified in any public reports or documents. All study staff completed human participants protection training. Participants were compensated an amount in Laotian kip equivalent to US $15 after completing each of the 2 primary assessments—at baseline and at the 12-week follow-up.

### Overall Study Design

**Figure 1:**
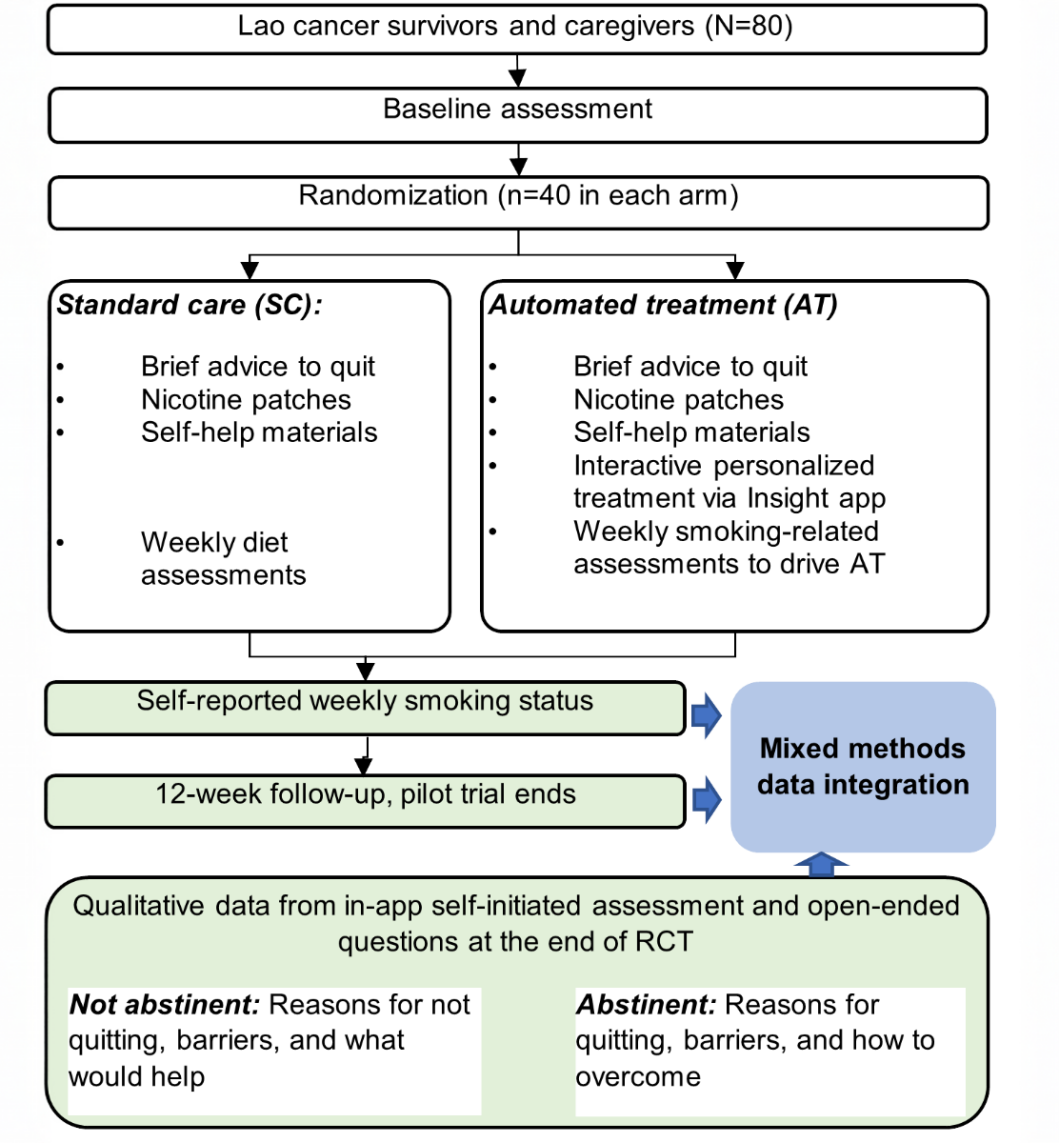
Study schema.

We used an intervention mixed methods research design, which included a pilot 2-group interventional RCT and an embedded qualitative component to explain RCT outcomes (Figure 1). In the pilot RCT, cancer survivors (n=30) or caregivers (n=50) of both sexes who smoked were recruited from SH and LNCC in Vientiane (the capital of Lao PDR). Recruited participants were randomized to 1 of 2 treatment groups: standard care (SC) or mHealth-based AT, delivered by our Insight app. Participants in both groups were asked to complete assessments delivered by REDCap (Research Electronic Data Capture; Vanderbilt University) at baseline and at the 12-week follow-up. All participants were also asked to complete brief weekly assessments (ecological momentary assessments) via the Insight app. SC consisted of brief advice to quit smoking delivered by research staff, self-help written materials, and a 2-week supply of nicotine replacement therapy (transdermal patches). AT consisted of all SC components plus our fully automated interactive smartphone-based treatment program, personalized and tailored to cancer survivors or caregivers, and delivered by our Insight app. Feasibility outcomes of interest include the percentages of intervention messages delivered and viewed (documented as digital stamps in our Insight app), usability, and participant retention at the 3-month follow-up. The preliminary efficacy outcome is biochemically confirmed self-reported 7-day point prevalence abstinence at 3 months post study enrollment. During the interventional RCT and at the 3-month follow-up assessment, we used additional open-ended questions to explore why and how the participants did or did not successfully quit smoking and stay abstinent.

### Conceptual Framework for AT

Our AT intervention was based on the Phase-Based Model (PBM), a theoretical framework that is specific to smoking cessation [21,22]. PBM partitions the cessation process into 4 phases: motivation, preparation (precessation), cessation (quit date to 4 weeks post quit), and maintenance (up to 6 months post quit); this RCT focuses on the latter 3 phases. PBM helps identify challenges/opportunities that smokers face at each phase, explains underlying phase-specific mechanisms, and facilitates selection of intervention components and measures. Using data from weekly assessments, our AT intervention dynamically targets several putative mechanisms, mainly those that are relevant across phases and have been reliably associated with long-term abstinence in previous studies: withdrawal/craving, motivation to quit, positive/negative affect, coping with stress and urges, and self-efficacy [22-26].

### Participants and Eligibility

#### Overview

**Figure 2:**
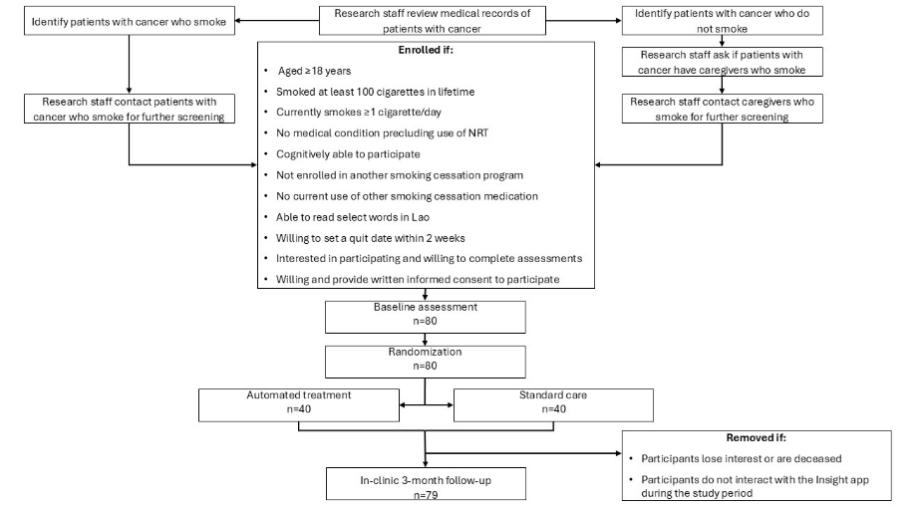
CONSORT flow diagram. CONSORT: Consolidated Standards of Reporting Trials; NRT: nicotine replacement therapy.

At SH, the Department of Oncology and Department of Breast and Gynecologic Cancer serve >200 patients with cancer each year. LNCC serves >400 new patients with various types of cancer each year [27]. Research staff reviewed medical records of patients with cancer receiving care at the 2 hospitals in the past 2 years, screened for their smoking status documented in the records, and contacted those who smoked for further screening. To recruit caregivers, research staff contacted random patients with cancer who did not smoke and asked if they had a caregiver and if the caregiver smoked. Given the pilot nature of this study, we proposed to recruit independent groups of cancer survivors and caregivers who smoked, that is, no dyads of smokers, to avoid potential interpersonal interactions. Participants who agreed to further screening were asked to complete a brief survey to determine their eligibility (Figure 2). REDCap was used to collect and manage information for most procedures, including screening eligible participants.

#### Inclusion Criteria

The pilot RCT included those who were (1) aged ≥18 years, (2) self-reported current combustible cigarette smokers (smoked at least 100 cigarettes in lifetime and currently smoke ≥1 cigarette/day), (3) willing to set a quit date within 2 weeks of study enrollment, (4) able to provide written informed consent to participate, and (5) able to read Lao (score of ≥4 points on the Rapid Estimate of Adult Literacy in Medicine—Short Form [28]).

#### Exclusion Criteria

Exclusion criteria were (1) history of a medical condition that precludes the use of nicotine patches, (2) ineligibility to participate based on medical or psychiatric conditions diagnosed by a physician/clinician, and (3) enrollment in another cessation program or current use of other cessation medications.

### Baseline Assessment and Randomization

Eligible individuals interested in participating completed an informed consent process and were enrolled in the study. Baseline assessments were conducted in person at the clinic. Enrolled participants completed a 45-minute baseline self-interview on tablets using the REDCap Mobile App. If a participant did not know how to use a tablet or preferred the questions to be read to them, research staff assisted them or conducted the interview. After the interview, research staff also assessed expired carbon monoxide (CO) with a CO monitor to verify smoking status, and collected additional data from participants’ medical records for AT personalization (eg, comorbidities or recent/current diagnosed health conditions). The tablets that contain the REDCap app were password-protected, encrypted, and could be remotely wiped if lost [29,30].

Participants were randomly assigned to SC or AT by the REDCap randomization module (simple, 1:1) and were blinded to their study group. Smartphones were loaned to participants as needed. All participants completed a brief training session on smartphone use that included downloading and navigating the Insight app. The Insight app also included a help button for instructions on how to use each feature, which participants could review at any time.

### Intervention Conditions

#### SC Intervention

SC consisted of brief advice to quit smoking delivered by research staff, self-help written materials (based on the WHO’s “A guide for tobacco users to quit” [31] that we translated to and validated in Lao), and a 2-week supply of nicotine patches. As mentioned above, there is no evidence-based smoking cessation support program implemented in Lao PDR to be used as standard care for our study. Thus, our SC treatment approach was based on a US guideline, which indicates that the use of nicotine replacement therapy doubles quit rates and should be considered the minimal standard care [32], together with the translated WHO’s behavioral guide.

#### AT Intervention

AT consisted of all SC components plus a fully automated smartphone-based treatment program that involved proactive, interactive, and personalized messages in Lao, along with some images. AT content was adapted from our previous efforts and was designed to target theoretical constructs of the PBM. That is, AT content was designed to increase motivation, self-efficacy, and the use of coping skills and reduce nicotine withdrawal symptoms and stress. In the R21 phase of the parent project in 2021-2022, we had culturally adapted the AT intervention content to Lao using the cultural adaptation of evidence-based interventions approach [33,34], as well as the International Quality of Life Assessment– and WHO-recommended methodology [35,36]. Briefly, major steps in the adaption and validation process included qualitative interviews with Lao health care providers and smokers to identify additional local factors that help smokers to quit, modifying the original English intervention content, forward translation, rating the quality of the translated materials on 5 dimensions (conceptual equivalence, clarity in meaning, comprehensibility, use of common simple language, and cultural appropriateness), conducting 4 focus groups with Lao smokers to evaluate the updated materials’ comprehensibility and cultural appropriateness, and backward translation for checking conceptual equivalence (particularly for PBM-based aspects). For this SurvLaos project, we further modified and validated the Lao intervention content with 10 patients with and survivors of cancer.

AT began immediately after enrollment and continued for 12 weeks (about 2-4 messages or images per day, delivered by our Insight app at participants’ preferred time). Quit date was set at day 14 post enrollment for both AT and SC participants. All daily messages were delivered as push notifications, and all necessary information and images were also available in the app for on-demand viewing. AT content was personalized and tailored to each participant’s baseline information (eg, sex, cancer types, caregiver status, and current health conditions), current PBM phase [21], and responses to the brief weekly assessments that drive AT. Insight enables complex built-in algorithms and branching logic, allowing us to create and deliver dynamically and individually tailored treatment content, using near-real-time input data from the weekly assessments. For example, there were different bins of messages for different cessation phases to ensure that the AT intervention targets critical mechanisms of each phase. Participants were asked to complete brief Insight-delivered assessments during each week of the AT treatment course, with questions about their smoking status in the past week, coping skills, and current levels of motivation and self-efficacy. Depending on participants’ responses to these questions (eg, quit or smoked, or low self-efficacy), different messages from different bins were delivered to the participants. Our AT only targeted cigarette smoking because it is the most common type of tobacco used by Lao smokers (95%) [37].

### Measures

#### In-Person Assessments at Baseline and at 12-Week Follow-Up

These were the primary assessments that collected 3 types of data: self-reported data collected by REDCap, expired air CO (assessed with Vitalograph BreathCO) to verify smoking status biochemically, and medical record data (for AT personalization).

#### Brief Weekly Smartphone Assessments

Participants in both groups were asked to complete brief weekly assessments via the Insight app on smartphones. AT participants received 6 PBM-based smoking-related questions; responses to these questions drove the tailored AT content (eg, tips to increase self-efficacy if participants indicated a low self-efficacy). To balance the effects of these weekly contacts between the RCT groups, we also asked SC participants to complete a weekly 6-item assessment; however, these questions were about diet. All participants were provided a prepaid 3-month unlimited data plan.

#### Outcomes

Feasibility outcomes of interest included Insight app use and adherence, usability, and participant retention at the 3-month follow-up. Measures of Insight app use and adherence, documented as digital stamps in our Insight app, included numbers of messages or images opened and viewed, weekly assessments opened and completed, and other activity logs documented in our Insight platform. Participants answered questions regarding Insight app usability and their satisfaction with the intervention at the 12-week follow-up assessment. Retention was documented by research staff.

The preliminary efficacy outcome was smoking status at 12 weeks post enrollment. Abstinence was defined as biochemically confirmed self-reported 7-day point prevalence abstinence with expired CO below 6 ppm. Secondary outcomes included continuous and sustained abstinence, number of quit attempts, and length of abstinence. There was also an in-app, participant-initiated qualitative assessment, with an app home screen button labeled “Share with us anything on your mind” and an open-text field for responses, to capture the participant’s thoughts, feelings, and behaviors.

#### Additional Open-Ended Questions

After the structured assessment for the RCT at the 3-month follow-up, we asked additional open-ended questions (Multimedia Appendix 1) to explore why and how the participants did or did not successfully quit smoking and stay abstinent. Specifically, for participants who were biochemically confirmed abstinent, the questions focused on what motivated them to quit and how they overcame challenges or barriers to stay abstinent. For participants who were not abstinent, the questions focused on why they did not or could not quit, what they underwent, what challenges or barriers they had, and what would help them quit in the future.

### Data Analysis Plan

#### RCT Outcome Analysis

Regarding the feasibility of AT, we hypothesize that ≥75% of AT content will be viewed/opened as indicated by digital date/time stamp in the Insight app. The 75% threshold is anticipated based on the observed rates in our similar pilot RCT in Cambodia, which showed that 81% of all delivered messages and assessments were opened and resulted in a statistically significant preliminary efficacy [20]. Descriptive statistics will be used to assess the feasibility outcomes of AT.

Regarding the preliminary efficacy of AT, we hypothesize that at the 12-week follow-up, 7-day point prevalence abstinence will be higher in the AT (vs SC) group. We will use log-binomial regression with an intent-to-treat approach (ie, missing=smoking) to compare the effect of the treatment on the dichotomous abstinence outcome at 12 weeks post enrollment, controlling for selected covariates such as sex, education level, and/or baseline stress level. Secondary outcomes will be examined using appropriate linear and generalized linear mixed models, with log binomial and linear regression for binary and continuous variables, respectively. Given the preliminary nature of the RCT, we do not expect to have sufficient power for full hypothesis testing. However, from our previous work, we anticipate that the abstinence at the 12-week follow-up will be 8% in the SC group [20]. With 40 participants in each treatment group, we will have 80% power to detect a difference as small as 20% in smoking abstinence rates between groups (α=.05, 1-tailed, unadjusted).

#### Mixed Methods Data Analysis

Two research team members will perform qualitative data coding with the aid of the R-QDA [38]. Discrepancies will be discussed to achieve intercoder agreement. Codes and quotations will be arranged into focused themes (ie, thematic content analysis) to develop the mixed methods data inventory [39]. Codes and themes will be based on the purpose of this component (ie, explaining the RCT preliminary efficacy outcome) and on theoretical constructs of the PBM. Next, we will examine quantitative (RCT) and qualitative data jointly, using the integration strategy of explaining with the intent to explore why participants could or could not quit and stay abstinent [39,40]. Mixed methods–linked data, results, and meta-inferences will be presented in visual joint displays [40], similar to our previous publication [20].

## Results

Participant enrollment started in April 2022 and ended in February 2023 (ClinicalTrials.gov ID: NCT05253573) with a final sample of 80 participants. Follow-ups were completed in May 2023, with a loss to follow-up due to the death of a patient with cancer. Implementation data in Insight and REDCap platforms were frequently analyzed to monitor research protocol fidelity. Outcome analyses are ongoing, and results are expected to be published in 2025.

## Discussion

### Anticipated Findings

In Lao PDR, tobacco smoking prevalence among patients with cancer is high, ultimately affecting cancer treatment outcomes. Nevertheless, there are no tobacco treatment programs in cancer hospitals or institutions in Lao PDR. Our study is the first effort that uses scalable and sustainable mHealth technology to provide tobacco cessation treatment to patients with or survivors of cancer and their caregivers in Lao PDR. The study adapted an evidence-based cancer survivorship care service (ie, smartphone-based smoking cessation treatment) in the United States to serve similar Lao populations. The results of this study will inform the feasibility, acceptability, and preliminary efficacy of AT in offering tobacco cessation support to Lao cancer survivors and caregivers. We hypothesize that the AT approach will be highly feasible and acceptable, as found in our previous studies. For example, in a previous pilot study with 50 Cambodian smokers, we found that most participants agreed that our mHealth-based treatment program was helpful in supporting smoking cessation (92%) and would recommend it to other smokers (88%) [20]. In another pilot study with 50 Lao patients with respiratory disorders, AT was highly acceptable: 91.6% agreed or strongly agreed that our app was easy to use, 100% of AT participants agreed or strongly agreed that the message content was easy to understand, and 95.8% felt comfortable with opening messages in public places [41]. For this SurvLaos project, one available result of the feasibility—the retention of participants through the 3-month study period—has been shown to be very promising (all surviving participants completed the 3-month follow-up). We also anticipate that the AT, compared to the SC, will demonstrate a higher preliminary efficacy as in our pilot study in Cambodia [20]. By demonstrating these critical metrics, this SurvLaos project will lay a foundation for a subsequent fully powered RCT to evaluate the actual efficacy of our mHealth-based AT program. Ultimately, the projects have the potential to improve cancer care and cancer survivorship research in Lao PDR and in other LMICs. In addition, their results will suggest the potential of a cancer care delivery model beyond health care settings (ie, an mHealth approach) that can be applicable to other behavioral interventions/treatments targeting cancer survivors and caregivers.

This course of research is conducted by a uniquely qualified multidisciplinary research team with extensive experience in conducting health promotion research in international settings and in mixed methods studies. US coinvestigators provide their valuable expertise in behavioral sciences, mHealth tobacco cessation research for low-income and other underserved populations, tobacco health risk communication, mHealth methodology, and biostatistics. This project has been implemented by the National Center for Laboratory and Epidemiology, a core unit of the Lao MOH that is responsible for a wide range of public health issues. Thus, our project can use several local expertise and resources for the successful implementation of the interventions in Lao PDR. In addition, the project is built on a strong partnership with stakeholders at other national institutions in Lao PDR, including SH, the LNCC, the NTCC, and the MOH. This partnership will enhance the intervention’s sustainability in the future.

Our proposed mHealth intervention approach is novel in many aspects. Our Insight platform enables ecological momentary assessments and just-in-time adaptive intervention for various health behaviors or issues. The use of Insight allows our AT to function autonomously and minimizes human involvement, making the approach affordable for large-scale implementation in LMICs. Using the friendly, intuitive Insight platform’s interface, Lao researchers can directly manage AT as they wish without knowledge of programming languages. Thus, in the subsequent implementation phase of AT or in future cancer survivorship research projects, Lao researchers can modify the AT content in Insight to target other behavioral, physical, and psychosocial survivorship outcomes (eg, monitoring cancer treatment compliance, fatigue, and complications; providing timely personalized screening recommendations to detect recurrence early; or improving retention in care).

This study has some limitations. First, this proposal was submitted to a small supplementary funding opportunity that supported pilot studies, and the data collection is expected to be completed within 1 year. Given the pilot nature of the project and the limited resources and time, a larger sample size or a longer follow-up period was not feasible. Second, the small sample size may not provide sufficient statistical power to evaluate fully the intervention’s efficacy or to perform subgroup analyses (eg, by sex, cancer types, or cancer stages). Thus, a future large-scale fully powered RCT is needed for these purposes. Finally, the use of mHealth and smartphone approaches may exclude some patients who do not own a smartphone or do not know how to use a smartphone. However, as we explained above, the trend in smartphone ownership in Lao PDR over the past decade has grown substantially, which clearly indicates that smartphone ownership will be nearly ubiquitous in the near future.

### Conclusion

Developing and evaluating a scalable and sustainable tobacco cessation treatment program for patients with and survivors of cancer and their caregivers in Lao PDR is a pressing need to improve cancer treatment and survivor outcomes. Our course of research aims to address this critical need. The preliminary data from this pilot project will lay a foundation for a subsequent fully powered RCT to evaluate the actual efficacy of our mHealth-based AT program. Ultimately, our course of research will contribute to reducing tobacco-related complications in cancer treatments, comorbidities, tobacco-related cancer recurrence, and mortality rates in Lao PDR.

## Appendix

Multimedia Appendix 1 Open-ended questions for the qualitative component.

## Abbreviations

AT: automated treatment
CO: carbon monoxide
IRB: institutional review board
Lao PDR: Lao People’s Democratic Republic
LMIC: low- and middle-income country
LNCC: Lao National Cancer Center
mHealth: mobile health
MOH: Ministry of Health
NCI: National Cancer Institute
NECHR: National Ethics Committee for Health Research
NTCC: National Tobacco Control Committee
OUHSC: University of Oklahoma Health Sciences Center
PBM: Phase-Based Model
RCT: randomized controlled trial
REDCap: Research Electronic Data Capture
SC: standard care
SH: Setthathirath Hospital
WHO: World Health Organization

## Data Availability

Data generated during this study will be available upon request to the corresponding author, following the NIH’s, OUHSC’s, and Lao Ministry of Health’s data sharing requirements.
