# Supplementary material for: Mobile Health Technology for Personalized Tobacco Cessation Support Among Cancer Survivors and Caregivers in Laos (Project SurvLaos): Protocol for a Pilot Randomized Controlled Trial
Source: JMIR Res Protoc. 2025 Jul 30;14:e66517. doi: 10.2196/66517 (PMC12351191; doi:10.2196/66517)
Supplement: Multimedia Appendix 1 [file resprot_v14i1e66517_app1.docx]

# In-app Participant-Initiated Assessment

| **#** | **English** | **Lao** |
| --- | --- | --- |
|  | Share with us anything on your mind | ​ແບ່ງປັນ​ກັບ​ພວກ​ເຮົາສິ່ງ​ໃດ​ກໍ່​ໄດ້​ທີ່ຢູ່​ໃນ​ໃຈ​ຂອງ​ທ່ານ |

# Open-ended questions after the trial follow-up structured assessment

| **#** | **English** | **Lao** |
| --- | --- | --- |
|  | ***[For quitters:***  *SS02=NO and CO<6ppm]* | ***[ສໍາລັບຜູ້ອອກຢາສູບ:***  *SS02=NO and CO<6ppm]* |
| QL01 | Please tell us what the main reasons for you to quit smoking were? [Probing] What really motivated you to quit smoking? _______ | ກະລຸນາບອກພວກ​ເຮົາ​ວ່າ​ເຫດຜົນ​ຫຼັກທີ່​ເຮັດ​ໃຫ້​ທ່ານອອກ​ຢາສູບ​ໄດ້ ​ແມ່ນຫຍັງ? ອັນ​ໃດ​ແທ້ໆ​ທີ່​ກະຕຸ້ນທ່ານ​ໃຫ້ອອກຢາສຸບ?_____________________ |
| QL02 | In the past 3 months, have you used any other things or methods to help you quit smoking? If yes, what did you use? ______ | ໃນຊ່ວງ 3 ເດືອນຜ່ານມາ, ທ່ານໄດ້ໃຊ້ສິ່ງ​ຂອງ ຫຼື ວິທີການອັນອື່ນ ​ເພື່ອ​ຊ່ວຍ​ທ່ານ​ອອກ​ຢາສູບບໍ່? ຖ້າ​ແມ່ນ, ທ່ານ​ໄດ້​ໃຊ້​ຫຍັງ? ____________________ |
| QL03 | Please tell us barriers or difficulties that you faced for quitting smoking? How did you overcome these? _______ | ກະລຸນາ​ບອກພວກ​ເຮົາ ອຸປະສັກ ຫຼື ຄວາມ​ຫຍຸ້ງຍາກທີ່​ທ່ານ​ປະ​ເຊີນໃນການອອກ​ຢາສູບ​ບໍ? ທ່ານເອົາຊະນະສິ່ງ​ເຫຼົ່າ​ນີ້ໄດ້ແນວໃດ? ____________ |
|  | ***[For smokers:***  *SS02=YES or CO≥6ppm]* | ***[ສໍາລັບຜູ້ສູບຢາ:***  *SS02=YES or CO≥6ppm]* |
| QL04 | Please tell us why you cannot quit smoking? [Probing] What are barriers or difficulties for you to quit smoking? _______ | ກະລຸນາ​ບອກພວກ​ເຮົາ ເປັນຫຍັງທ່ານຈື່ງບໍ່ສາມາດອອກຢາສູບໄດ້? ອັນ​ໃດເປັນອຸປະສັກ ຫຼື ຄວາມຫຍຸ້ງຍາກສຳລັບທ່ານທີ່​ຈະ​ອອກຢາສູບ______________ |
| QL05 | In the past 3 months, have you used any other things or methods to help you quit smoking? If yes, what did you use? ______ | ໃນຊ່ວງ 3 ເດືອນຜ່ານມາ, ທ່ານໄດ້ໃຊ້ສິ່ງ​ຂອງ ຫຼື ວິທີການອັນອື່ນ ​ເພື່ອ​ຊ່ວຍ​ທ່ານ​ອອກ​ຢາສູບບໍ່? ຖ້າ​ແມ່ນ, ທ່ານ​ໄດ້​ໃຊ້​ຫຍັງ? ____________________ |
| QL06 | For what reasons would you stop smoking in the future? [Probing] What do you think that would really motivated you to quit smoking? _______ | ດ້ວຍ​ເຫດຜົນ​ອັນ​ໃດ​ທີ່ທ່ານຈະ​​ເຊົາ​ຢາສູບ​​ໃນ​ອະນາຄົດ? ທ່ານ​ຄິດ​ວ່າ ອັນ​ໃດ​ແທ້ໆ​ທີ່ຈະກະຕຸ້ນທ່ານ​​ອອກ​ຢາສູບ?___________ |
|  | ***[For all participants]*** | ສໍາລັບຜູ້ເຂົ້າຮ່ວມທຸກຄົນ |
| QL07 | Please tell us things that should be changed to improve the smoking-cessation treatment program that you have received in the past 3 months | ກະລຸນາບອກພວກເຮົາເຖິງ ​ສິ່ງ​ທີ່​ຄວນ​ປ່ຽນ​ແປງ ​ເພື່ອປັບປຸງ​ແຜນ​ງານບຳບັດການອອກ​ຢາສູບທີ່ທ່ານໄດ້ຮັບ ໃນຊ່ວງ 3 ເດືອນທີ່ຜ່ານມາ. |
